# A new species of *Coespeletia* (Asteraceae, Millerieae) from Venezuela

**DOI:** 10.3897/phytokeys.28.6378

**Published:** 2013-11-04

**Authors:** Mauricio Diazgranados, Gilberto Morillo

**Affiliations:** 1 Dept. of Botany, MRC 166, National Museum of Natural History, P.O. Box 37012, Smithsonian Institution, Washington D.C. 20013-7012, United States; 2 Departamento de Botánica, Escuela de Ingeniería Forestal, Facultad de Ciencias Forestales y Ambientales, Universidad de Los Andes, Mérida 5101A, Venezuela

**Keywords:** *Coespeletia*, Compositae, Espeletiinae, frailejón, Millerieae, Páramos, Venezuela

## Abstract

A new species of *Coespeletia* from the páramos of Mérida (Venezuela) is described here. This species, named *Coespeletia palustris*, is found in a few marshy areas of the páramo. It is closely related to *C. moritziana*, but differs from it in a smaller number of florets in the capitula, larger ray flowers with longer ligulae and longer linguiform appendages, smaller pollen grains, larger cypselae, ebracteate scapes, leaves and inflorescences with more whitish indumentum, larger leaf sheaths, and marshy habitat.

## Introduction

The genus *Coespeletia* Cuatrec. (Espeletiinae: Asteraceae) was described based on its racemiform monochasial inflorescences, sometimes reduced to a monocephalous scape, with capitula semiglobose or patelliform, and ray flowers usually not exceeding the involucres. Later palynological studies supported this genus as a clade. Most of the Espeletiinae species have the *Aspilia*-pollen type, but the pollen type of *Coespeletia* is unique, called the *Coespeletia*-type (Salgado-Labouriau 1982; Cuatrecasas 2013). Pollen grains of this type have a larger number of equatorial spines (≥16), the spines are much shorter (≤4 µm), and have smaller spine length/polar diameter ratios (<0.15) than the rest of the Espeletiinae (Cuatrecasas 2013). These pollen characteristics may represent a particular adaptation to wind pollination at high-elevation habitats (Cuatrecasas 2013), where most of the species of *Coespeletia* are found (Diazgranados 2012).

Currently there are seven described species of *Coespeletia*: six endemic to the Andes of Venezuela (*Coespeletia albarregensis* Cuatrec., *Coespeletia elongata* (A. C. Sm.) Cuatrec., *Coespeletia moritziana* (Sch. Bip. ex Wedd.) Cuatrec., *Coespeletia spicata* (Sch. Bip. ex Wedd.) Cuatrec., *Coespeletia thyrsiformis* (A. C. Sm.) Cuatrec. (including *Coespeletia thyrsiformis* f. *marcana* (Cuatrec.) Cuatrec.), and *Coespeletia timotensis* (Cuatrec.) Cuatrec.), and one species recently discovered in northern Colombia (*Coespeletia laxiflora* (S. Díaz & Rodr.-Cabeza) S. Díaz & Rodr.-Cabeza) (Diazgranados 2012). However, the latter species is under re-evaluation by the first author. The Venezuelan species grow in the páramos of Sierra Nevada de Mérida, Sierra Nevada de Santo Domingo and Sierra del Norte in the state of Mérida, and in the Páramos de la Negra, del Batallón and del Zumbador in the state of Táchira (Diazgranados 2012; Cuatrecasas 2013). Most of the species are restricted to very high elevations, in a range between 3800–4800 m (Diazgranados 2012). Only one species can grow below 3000 m, six species can be found at 3800 m, and five species are adapted to superpáramos at elevations above 4000 m. The highest elevation ever reported for any Espeletiinae specimen is 4780 m, for *Coespeletia timotensis* (coll. *L.Ruiz-Terán 851*). However, according to Cuatrecasas (2013), *Coespeletia moritziana* can grow above 4800 m, on rocky crests emerging from glacial blocks.

Even after decades of studies and collections in the páramos, numerous localities remain unstudied, and there are still several taxonomic problems and interesting challenges within the Espeletiinae (Diazgranados 2012). The new species described in this paper is called “palustris” because of the marshy habitat in which it grows. High-elevation marshes and wetlands are among the ecosystems which are most impacted by climate change (Rabatel et al. 2013, Vuille 2013). Therefore this species may be at a certain risk of extinction as well.

## Methods

Material of the new species was collected in the field in 2011. Two expeditions were organized to the native habitat, and collections were preserved and distributed to the herbaria MER and VEN. Photomicrographs were taken by the first author at the Scanning Electron Microscopy Laboratory of the National Museum of Natural History, in Washington DC. In addition, numerous collections already present in several herbaria were studied (see ‘Specimens examined’ section below, with specimens listed by geographic location, including specimens for *Coespeletia moritziana* and *Coespeletia palustris*). Illustrations were done by Lauren Merchant, from Saint Louis University.

## Taxonomy

### Coespeletia palustris

M. Diazgranados & G. Morillo sp. nov.

urn:lsid:ipni.org:names:77133498-1

http://species-id.net/wiki/Coespeletia_palustris

Figs 1 –4

#### Diagnosis.

Related to *Coespeletia moritziana* but differs in indumentum primarily whitish, larger leaf sheaths, proximal portion of young leaves white, thicker scapes, which are ebracteate, larger phyllaries with pubescence unkempt, capitula with fewer flowers, which are much larger, and smaller pollen grains.

#### Type.

Venezuela, Mérida, Páramo de Santo Domingo, alrededores de la Laguna de los Patos, en zona de turbera. Alt. 3729 m., 8.77522 N, -70.80349 E, 29 September 2011, *G. Morillo*, *M. Diazgranados*, *L. Gámez*, *S. Rodríguez & J. Parra 14155* (holotype: MER, isotype: VEN). Additional collections from the type locality (paratypes): *L. Gamez*, *G. Morillo*, *J. Parra & S. Rodríguez 1097*, *1100* (MER).

#### Description.

Acaulescent (or subsessile) rosette of 60–80(–100) cm (including inflorescences) and of 40–60 cm in diameter; numerous inflorescences monocephalous, twice longer than leaves, with naked axes. Rosettes whitish, densely covered by whitish lanose indumentum, with little accumulation of marcescent leaves at the base. Leaves linear, with laminae (28–)32–36(–38) cm long, (0.7–)0.8–1.2(–1.3) cm wide, length to width ratio (28–)30(–35):1, thick, subsessile, with a pseudopetiole greenish and almost glabrous, 2 cm long and 0.5 cm wide. The leaves are covered by a dense lanose indumentum, grey-whitish. When young, leaves are golden yellow in the distal portion, and white in the proximal portion. The sheath is oblong, adaxially glabrescent and greenish or whitish, abaxially reddish-brown and slightly covered by hairs, (7–)7.5–9.5(–10) cm long, (2–)2.2–2.5(–2.7) cm wide.

Inflorescences numerous (>30), coetaneous, axillary, aphyllous, monocephalous, with a single nodding capitulum. Scape about twice longer than leaves, 60–80 cm long, 1.0–1.4 cm in diameter, erect, densely lanate, yellowish, relatively thick and stout, totally naked, without leafy bracts at the base.

Capitula radiate, patelliform, nodding, 4.5–6 cm in diameter including the ligules of the ray flowers; ligular circle well developed, as large as the involucre; discs (without counting ligular circle) 2.2–2.4 cm in diameter. Involucre sub-hemispherical, densely lanate, the pubescence unkempt, yellowish or whitish. Phyllaries pluriseriate, narrow and curved (almost curly), the outer ones 18–20 mm × 4.0–4.2 cm, linear to narrowly-lanceolate, becoming progressively shorter towards the inner-most phyllaries, 13–14 mm × 2.8–3.2 mm, only apically hairy.

Ray flowers ligulate, 95–200 per capitulum, in 3–5 rows. Corolla abaxially dark pink, adaxially yellowish becoming brown-reddish towards the apex, carnosulous, 12–14.2 mm long; ligules elliptical or oblong, bi- or tridentate, with 6 reddish-purple conspicuous nerves. Tube glanduliferous, 0.6–1.0 mm in diameter and 3.8–4.8(–6.0) mm in length, dark pink, the glands pediculate capitate, 150–160 μm long × 30–35 μm in diameter; tubes with 2 linguiform appendages 1.5–2.2 mm long; ovary whitish when young, becoming red in the extremes. Paleas 12–16(–17.5) mm long, 2.7–3.0 mm wide, brownish, with 3 main nerves. Styles 8–9 mm long, 0.2–0.3 mm wide, with branches about (1.7–)2.0–3.2 mm long, papillose. Cypselae oblong, triangular, 3.6–3.9 mm × (1.6–)1.7–2.3 mm.

Disc flowers 215–280, (8–)9–10 mm long (not counting the anthers), of purple appearance; corolla 5–5.5 mm long, reddish, yellowish pink or translucid in the lower half, lobes 5, 1–1.1 mm long and 0.5–0.6 mm wide at the base; tube glanduliferous, 0.8 mm wide × 2.6–4.5 mm long; anthers purple, sometimes exceeding the corolla by 2 mm, slightly translucid; paleas 9.8–10.2 mm long, 1.5–1.7 mm wide, brownish, with 3 main nerves.

Pollen yellow when fresh, tricolporate, 22.1–23.7 μm in equatorial diameter (not including spines), 25–26 μm in polar diameter; spines 110–116 total, equatorial spines 18, 2.3–3.2 μm long, slightly curved.

**Figure 1.**
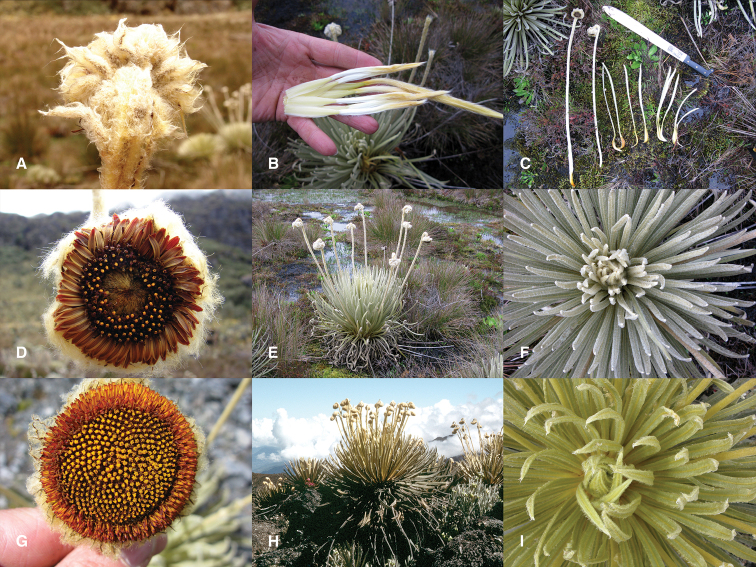
*Coespeletia palustris*: **A** abaxial view of the involucre **B** young leaves showing white basal portion **C** parts of the collection **D** capitulum of the holotype collection (Morillo et al. 14155) **E** acaulescent (or subsessile) habit and marshy habitat **F** whitish rosette from top. *Coespeletia moritziana*: **G** capitulum **H** caulescent habit and rocky habitat **I** yellowish rosette from top.

**Figure 2.**
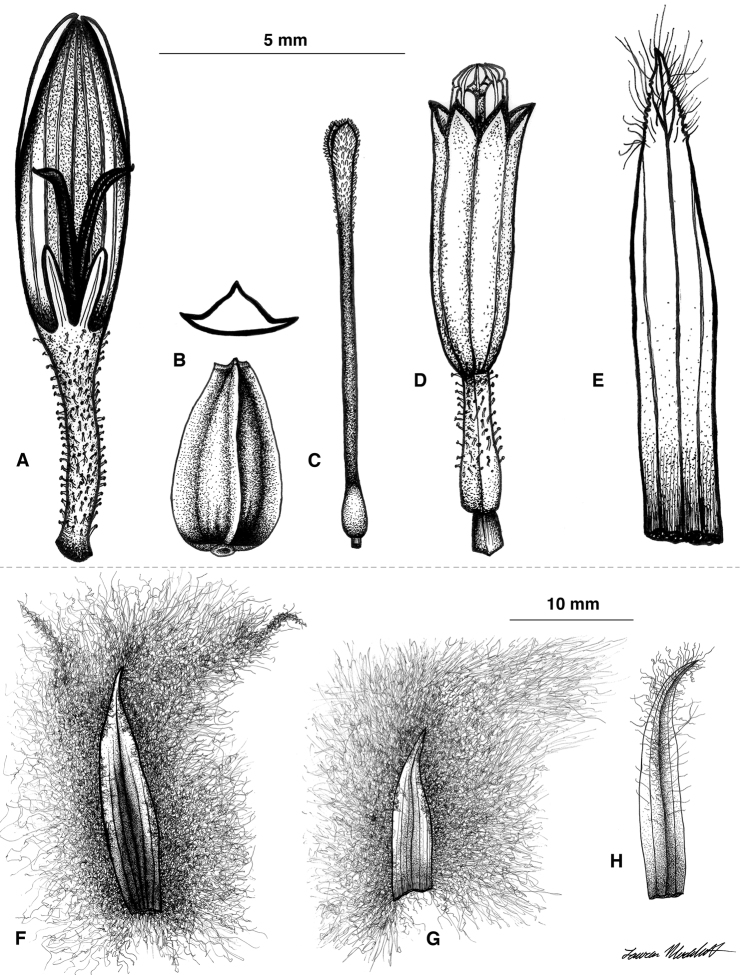
Illustrations of *Coespeletia palustris*. **A** Ray flower **B** cypsela **C** disc flower style **D** disk flower **E** disk flower palea **F** outer phyllary **G** inner (sterile) phyllary **H** fertile ray flower palea.

**Figure 3.**
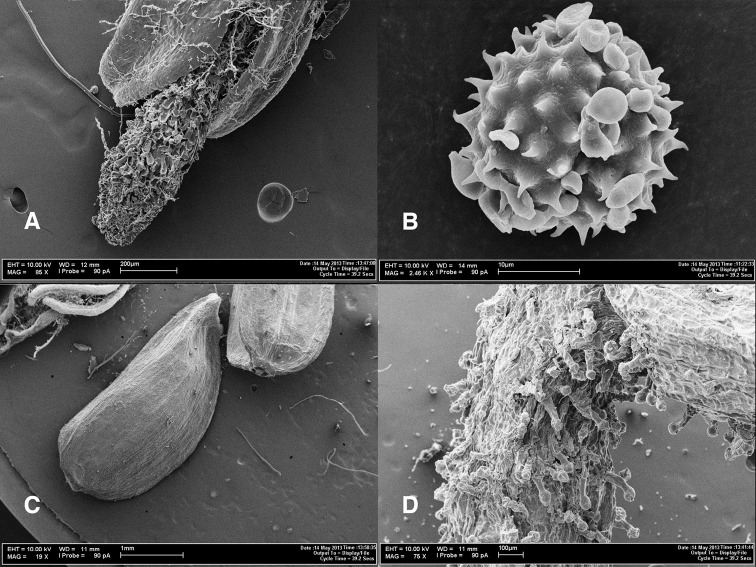
Photomicrographs of *Coespeletia palustris*. **A** Tip of stigmatic branches in a disc flower **B** pollen grain **C** cypsela **D** granduliferous tube of ray flower.

#### Distribution

**Figure 4.**
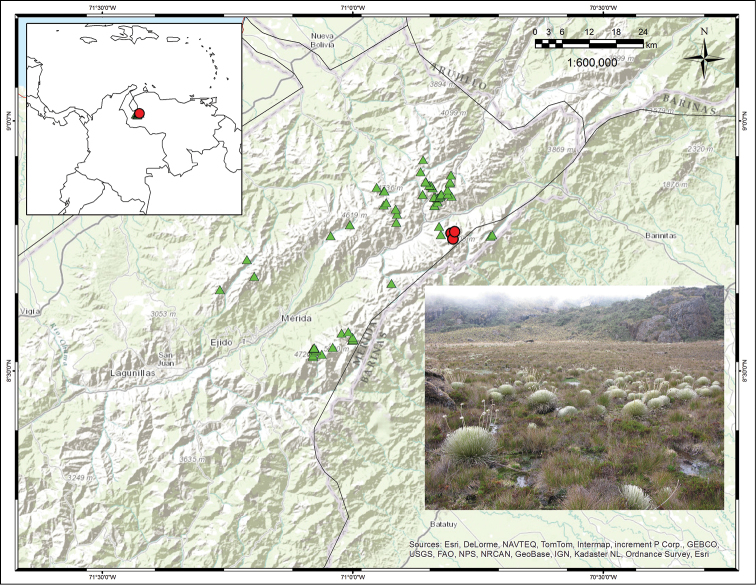
Distribution map showing collections for *Coespeletia palustris* (red circles) and *Coespeletia moritziana* (green triangles). Photograph of marshes around Laguna de los Patos, with a population of *Coespeletia palustris*.

(Fig. 4). Endemic to Venezuela. This species has been found in a few marshy areas of the Páramo de Santo Domingo (e.g. around the Laguna de los Patos) and the Sierra de la Culata, always in small areas of less than 0.5 km^2^.

#### Ecology.

Small population of about 200–400 individuals growing in marshes, on very swampy and wet soil. Other Espeletiinae found on the proximate drier slopes are: *Coespeletia timotensis*, *Espeletia schultzii* Wedd., *Espeletia weddellii* Sch. Bip. ex Wedd., and *Libanothamnus neriifolius* (Bonpl. ex Humb.) Ernst.

#### Etymology.

The name “palustris” is given because of the boggy habitat in which this species is found.

#### Conservation status.

This species may be at a certain risk of extinction, since it is found in a very particular habitat, sensitive to climate change.

#### Specimens examined.

*Coespeletia moritziana*: VENEZUELA. Mérida: Sierra Nevada de Mérida: 4000 m, 1842, *Linden 398* in part (isolectotype; BR, P, US!); Sierra Nevada, flower, Sep-Dec, *Moritz 1416* (F!); Páramo de Mucuchíes around Pico del Aguila, 4300 m, in Apartaderos, *Steyermark 55883* (F!, NY, VEN); id., 4150 m, Sep 1952, *Humbert 26458* (P, US!); id., Pico del Aguila, 4100 m, Apr 1952, *Gines 4794* (US!); id., 4200 m, 5 Oct 1969, *Cuatrecasas*, *Ruiz-Terán & Lopez-Figueiras 28031* (MERF, US!); id., 4000 m, 5 Dec 1936, *Chardón 5007* (COL!, US!); id., 20 Apr 1946, *Burkart 16802* (US!, VEN); id., Apartaderos towards Timotes, 3 Nov 1976, *Bernardi*, *Charpin & Jacquemoud 17064* (G, US!); id., 4118 m, 21–26 Apr 1959, *Barclay & Juajibioy 9649* (US!); id., 4200 m, 15 Nov 1975, *Badillo & Páez 6857* (MY, US!); id., 4000 m, 26 Jan 1939, *Alston 6631* (GH, NY, P, S, U, US!); Sierra Nevada above Mérida, Loma Redonda, 4000–4045 m, 14 Oct 1969, *Cuatrecasas*, *Ruiz-Terán & López-Figueiras 28093* (MERF, US!); id., 4050 m, 9 Dec 1983, *Sobrevila 1552* (US!); id., around Laguna Verde, below Pico Humboldt, 4100 m, 26 Feb 1971, *Ruiz-Terán & Lopez-Palacios 1617* (MERF, US!); id., Laguna Verde, 4000 m, on rocky slope, 4 Dec 1959, *Barclay & Juajibioy 10043* (US!); between Laguna Coromoto and Laguna Verde, 3800 m, Oct 1956, *Aristeguieta 2610* (F, NY, US!, VEN); Páramos around Pico Bolivar and Pico Espejo on trail to Nevados, 4150 m, 15–18 Dec 1959, *Barclay & Juajibioy 10224* (US!); id., Cañada of Laguna de Los Anteojos, 3930–3900 m, 22 Feb 1973, *Cuatrecasas*, *Ruiz-Terán & López-Figueiras 28584* (MERF, US!); Pico Toro, southern slope on the way to Los Nevados, frequently humid and marshy places, 4000–4200 m, 22 Jan 1972, *Ruiz-Terán 6882* (MERF, US!); Sierra Nevada de Santo Domingo; NW slopes, 4000–4400 m, 24–26 Sep 1952, *Humbert 26479* (COL, P, US!); Páramo de Laguna Brava, 3300 m, 22 May 1971, *López-Figueiras 8721* (MERF, US!); Páramo de Timotes, 3800–4200 m, 24 Jan 1928, *Pittier 12729* (F!, M, MO!, NY, VEN); id., 4200 m, 21 Jan 1922, *Jahn 869* (GH, K, M, NY!, US!, VEN); id., 3000–3500 m, 6 Dec 1910, *Jahn 150* (US!, VEN); id., Cabeceras of Quebrada El Turmero, afluent of Rio Motatan, 4260 m, in marshy places, 12 Nov 1984, *Berry 4402* (US!); between Timotes and Laguna Huacha, 3850 m, 21–26 Nov 1959, *Barclay & Juajibioy 9853*, 9889 (US!); Sierra del Norte west of La Culata: Alto de Piedras Blancas, vert N, in marshy spots, 17 Jul 1970, *Ruiz-Terán & López-Figueiras 369* (MERF, US!); id., 4100 m, 23 Nov. 1976, *Quintero 1697* (MER!), *1698* (MER!); Llano de Piedras Blancas, 4150–4200 m, 16 Jul 1970, *Ruiz-Terán & López-Figueiras 331* (MERF, US!); id., Páramo de Los Conejos in Zanjas de Los Castillos, 3800 m, 24 Mar 1972, *Ruiz-Terán 6975* (MERF, US!); id., in Indio Dormido, 3750 m, 23 Jul 1980, *López-Figueiras 23688* (MERF, US!); id., 3500 m, 1933, *Jahn 235* (US!); id., 24 Sep 1938, *Hanbury-Tracy 155* (K, NY, US!). *Coespeletia palustris* (paratypes): VENEZUELA. Laguna de Los Patos, 4000 m, 23 Nov 1959, *Barclay & Juajibioy 9741* (US!); id, 6 Feb 1966, *Schulz 252* (MER!); Páramo de Los Granates, Cañada del Padre, 3550 m, 10 Oct 1969, *Cuatrecasas*, *Ruiz-Terán & López-Figuieras 28057* (MERF, US!); id., 3480 m, 10 Oct 1969, *Cuatrecasas*, *Ruiz-Terán& López-Figuieras 28055* (MERF, US!); Páramo de Mucubaji, 6100 m, 6 Jun 1952, *Gines 4801* (US!); id., 3700 m, Sep 1956, *Aristeguieta 2445* (MER!, US!, VEN); id., 4670 m, 17 Nov 1958, *Buza 107* (F!, MER!); id., Laguna Negra, 18 May 1952, *Vareschi & Pannier 1019* (MER!); id., Cañada of Laguna de Los Anteojos, 3930–3900 m, 22 Feb 1973, *Cuatrecasas*, *Ruiz-Terán & López-Figueiras 28584* (MERF, US!); Páramo de Mucuñuque, 4100 m, 20 June 1958, *Buza 109* (MER!).

## Discussion

This species is closely related to *Coespeletia moritziana*. However, the habitat of *Coespeletia moritziana* is a dry rocky crest of superpáramo, while *Coespeletia palustris* grows in swampy grassy areas. As recognized by Cuatrecasas (2013), *Coespeletia moritziana* is highly polymorphic, with two main “forms”: the first, which includes the type collection, with thin scapes often with sparse sterile bracts; and the second, with much thicker scapes, naked, larger capitula, and longer ray corollas. Here we separate the second “form” observed by Cuatrecasas as the new species *Coespeletia palustris*, which grows in marshy areas, in contrast with the typical *Coespeletia moritziana*, adapted to rocky crests of higher elevations.

Hybridization is quite common between Espeletiinae species (Diazgranados 2012). For instance, there are three hybrids reported for *Coespeletia moritziana*: *Espeletia* × *aurantia* Aristeg. (*Coespeletia moritziana* × *Espeletia schultzii*); *Coespeletia moritziana* × *Coespeletia timotensis*; and *Espeletia schultzii* × *Coespeletia moritziana* (Diazgranados 2012). However, the populations of *Coespeletia palustris* are morphologically homogeneous, and rather different from the populations of *Coespeletia moritziana* seen in the rocky and colder superpáramos. Despite the high polymorphism of *Coespeletia moritziana*, there are several stable morphological differences between *Coespeletia moritziana* and *Coespeletia palustris*. Some of those differencesseem to correspond to adaptation to their distinct habitats. For instance, an increase in the size of capitula and the number of florets seems to be an adaptation to the cryothermal zone (in *Coespeletia moritziana*), while the thicker leaves with longer sheaths can be correlated with marshy habitats (in *Coespeletia palustris*). Still, the polymorphic *Coespeletia moritziana* ‘complex’ will require additional taxonomical work in the near future.

### Key to differentiate *Coespeletia palustris* and *Coespeletia moritziana*

**Table:** 

1	Rosette sometimes caulescent (stem up to 60 cm), of yellowish appearance; leaves with laminae 25–48 cm long, rather thin, with sheaths 5–7 cm × 0.9–2.2 cm; proximal portion of young leaves generally golden or reddish-golden; scapes often bracteate, thin; outer phyllaries 3.0–1.8 mm wide; ray flowers 400–738 per capitulum, 6.5–9(16) mm long, shorter than involucre, with styles 4.5–7.0 mm long and linguiform appendages 1.0–1.5(–3.0) mm long; cypselae (2.5–3.0(–5.0) mm × 1.8–2.0(–2.5) mm); disc flowers 614–862 per capitulum, 9–10 mm long; pollen 24.5–29.8 μm in equatorial diameter, and 28–35 μm in polar diameter	*Coespeletia moritziana*
–	Rosette always acaulescent (or subsessile), of whitish appearance; leaves with laminae 28–38 cm long, rather thick, with sheaths (7–)7.5–9.5(–10) cm × (2–)2.2–2.5(–2.7) cm; proximal portion of young leaves white; scapes ebracteate, thick; outer phyllaries 4.0–4.2 mm wide; ray flowers 95–200 per capitulum, 12–14 mm long, sometimes equaling the involucre, with styles 8–9 mm long and linguiform appendages 1.5–2.2 mm long; cypselae (3.6–3.9 mm × (1.6–)1.7–2.3 mm); disc flowers 215–280 per capitulum, 6.5–8(11) mm long; pollen 22.1–23.7 μm in equatorial diameter, and 25–26 μm in polar diameter	*Coespeletia palustris*

## Supplementary Material

XML Treatment for Coespeletia palustris
